# Moderating role of 1-minute abdominal test in the relationship between cardiometabolic risk factors and adiponectin concentration in adolescents

**DOI:** 10.1186/s12887-024-04554-z

**Published:** 2024-01-23

**Authors:** Maiara Cristina Tadiotto, Patricia Ribeiro Paes Corazza, Francisco José de Menezes-Junior, Tatiana Aparecida Affornali Tozo, Frederico Bento de Moraes-Junior, Caroline Brand, Kátia Sheylla Malta Purim, Jorge Mota, Neiva Leite

**Affiliations:** 1 Department of Physical Education, Federal University of Paraná, Street Col. Francisco H. dos Santos, 100, Jardim das Americas, Curitiba, Paraná 81531-980 Brazil; 2 Physical Education School, IRyS Group, Pontificia Universidad Católica de Valparaíso, Valparaíso, Chile; 3 Department of Medicine, Positive University, Curitiba, Brazil; 4 Faculty of Sport, University of Porto, Porto, Portugal

**Keywords:** Adiposity, Adipokines, Risk factors, Muscular fitness, Physical fitness, Youth

## Abstract

**Background:**

Adiponectin is an anti-inflammatory cytokine secreted by adipose tissue, has been associated with adiposity and cardiometabolic risk, and has controversial results with muscular fitness. The aim of this study was to analyze the interaction of 1-minute abdominal test in the relationship between adiposity, body composition, cardiometabolic risk and adiponectin concentration in adolescents.

**Methods:**

This is a cross-sectional study conducted with 62 adolescents of both sexes, aged 11 to 16 years, approved by the Ethics Committee of Research in Humans (CAEE: 62963916.0.0000.5223). Body mass, height, abdominal circumference (AC), waist circumference (WC), fat mass (FM), fat-free mass (FFM), high density lipoprotein (HDL-c), low density lipoprotein (LDL-c), triglycerides (TG), adiponectin, systolic blood pressure (SBP), diastolic blood pressure (DBP) and mean blood pressure (MBP), 1-minute abdominal test (ABD) were measured. Body mass index (BMI), z-score BMI (BMI-z), triponderal mass index (TMI), and waist-to-height ratio (WHtR) were calculated. The macro PROCESS for SPSS v.24.0 was used for moderation analyses, with linear regression models.

**Results:**

Inverse interactions were found for adiposity (BMI, BMI-z, TMI, AC, WC, WHtR), body composition (FM, FFM) and CMRF (SBP, DBP, MBP, TG) versus 1-minute abdominal test with adiponectin concentration, demonstrating that abdominal test is a moderator in these relationships.

**Conclusion:**

We conclude that 1-minute abdominal test may play an important role in the relationship between obesity and cardiometabolic risk. We found that muscular fitness can confer a protective effect on adolescents with high levels of abdominal test.

## Background

Adiponectin was originally identified as a protein expressed and produced by adipocytes by four independent research groups [1–4]. It is the only adipocytokine whose plasma concentration decreases in obese individuals, which is paradoxical, since it is secreted by adipose tissue [5, 6]. Unlike other substances in adipose tissue, it has anti-inflammatory and anti-atherogenic properties [7, 8], acting as a protective factor for cardiovascular diseases, in insulin sensitization, in reducing the inflammatory response and in atherogenesis [9].

Excessive accumulation of adiposity is associated with secretion of inflammatory cytokines [10] and reduction of anti-inflammatory, such as adiponectin [11]. In addition, has been associated with insufficient levels of physical activity, increased time in sedentary activities and inadequate nutrition, factors that have led to low physical fitness [12] and the appearance of comorbidities. In pediatric population, low adiponectin concentration it’s associated with higher adiposity and metabolic risk and has controversial results with physical fitness [13–15].

Current literature provides consistent results regarding the association between cardiometabolic risk factors (CMRF) and cardiorespiratory fitness [16]. Recently, there has been great interest in verifying the independent effects of muscular fitness (MF) on physical and metabolic health in children and adolescents [17]. Several studies have sought to verify the influence of physical fitness on cardiometabolic health [13, 16], and adiponectin has aroused interest, presenting itself as a potential marker of cardiometabolic protection [6].

Relationships between adiposity, CMRF and adiponectin concentration are described in the scientific literature [14], as well as the interaction between adiposity, physical fitness and their health outcomes in adolescents has been investigated [18]. Paradoxically, investigations have shown that adiponectin is inversely associated with MF, while studies have found an inverse relationship [13, 15], others found no relationship [19, 20]. This evidence may lead to a complex analysis that other factors may influence and modify the relationship between adiponectin and MF, while muscular fitness may interact by moderating the relationship between adiposity and metabolic process [18]. Therefore, our aim was to analyze the interaction of abdominal test in the relationship between adiposity, body composition, CMRF and adiponectin concentration in adolescents.

## Methods

### Study design and population

The present study, of a correlational descriptive character with cross-sectional design, was approved by the Ethics Committee of Research in Humans UniDBSCO University Center (CAEE: 62963916.0.0000.5223). Population consisted of adolescents from Curitiba and the Metropolitan Region - Paraná/Brazil and the recruitment was carried out in a non-probabilistic sampling process, for convenience. Parents and/or guardians were informed about the research procedures and signed the free and informed consent form, as well as their adolescents. Data collection was carried out between March and April 2019.

The sample size was calculated a priori using the G*Power software (v.3.1.9.2), through the linear multiple regression, with three predictive variables. A power of 0.95, a of 0.05, and effect size (f) of 0.20 were assigned. Based on these criteria, the minimum sample size was 56 participants. The study included 62 adolescents, both sexes, aged between 11 and 16 years.

Inclusion criteria were: (a) agree to participate in all assessments; (b) consent form signed by the parents and/or guardians; (c) not present contraindication for carrying out the tests; and (c) not use drugs that interfere with research results. Exclusion criteria were: (a) muscle injury or physical limitation that could interfere with the performance in any of testing procedures; (b) participation in other activities that interfered with the research results; and (c) not participate in all measurements.

### Somatic maturation and anthropometric measures

Estimated by determining the distance in years from peak height velocity by the mathematical model based on height, age, and sex. The prediction of age at peak height velocity (APHV) was determined by subtracting from the chronological age [21].

Measurements were performed the according to the procedures described in the literature [22]. Height was measured using a portable stadiometer with an accuracy of 0.1 cm and body mass was measured using a digital reading platform scale, previously calibrated, with a maximum capacity of 200 kg and accuracy of 0.1 kg. Abdominal circumference (AC) and waist circumference (WC) measurements were evaluated with a flexible and inextensible tape with a resolution of 0.1 cm. Body mass index (BMI) and z-score BMI (BMI-z) were calculated in the WHO Anthro Plus® [23]. Triponderal mass index (TMI) was calculated as the ratio between body mass and height cubed. Waist-to-height ratio (WHtR) was calculated by the quotient between WC and height.

### Body composition

Body composition was assessed using tetrapolar bioelectrical impedance analysis (Biodynamics®), was performed in the morning, with those evaluated in the supine position for about ten minutes. Previously instructed: (a) abstain from food and drinks the past 12 h; (b) avoid vigorous physical efforts the past 12 h; (c) abstain from of alcohol and caffeinated drinks over the past 48 h; (d) not using diuretics over the past seven days; (e) urinate about 30 min before the exam; and (f) do not use metallic objects. Fat-free mass (FFM) and fat mass (FM) were calculated.

### Clinics and metabolic variables

Systolic blood pressure (SBP) and diastolic blood pressure (DBP) were measured using an aneroid-type mercury sphygmomanometer, previously calibrated, with the appropriate cuff size for the arm circumference, after ten minutes of rest, with the adolescent sitting and right arm supported at heart level. Mean blood pressure (MBP) was calculated [24].

Blood samples were collected in the morning by specialists using standard techniques after twelve hours of fasting for triglycerides (TG), high density lipoprotein cholesterol (HDL-c), low density lipoprotein cholesterol (LDL-c) and adiponectin concentration. Colorimetric enzymatic method was used to measure TG, HDL-c, and LDL-c. Adiponectin concentration by the ELISA method, according to the specifications of the total adiponectin concentration.

### 1-minute abdominal test

Localized muscular resistance was measured using the 1-minute abdominal test, in which the adolescent was positioned in the supine position, with knees flexed at 45º, feet flat on the floor and arms crossed and supported at chest height. The evaluator, with his hands, holding the ankles, fixing them to the ground and to the signal, the adolescent started the trunk flexion movements until touching the elbows on the thighs, returning to the initial position, performing the maximum of complete repetitions in 1-minute [25].

### Statistical analysis

Data normality was verified the Kolmogorov-Smirnov test and the assumption of homogeneity of variance was evaluated using Levene’s test. Standard descriptive statistical procedures using means and standard deviations were used to characterize the sample. For comparisons, analysis of covariance with post-hoc Bonferroni was used. Moderation analyses were tested by linear multiple regression models. Adiponectin was considered the dependent variable; adiposity, body composition and CMRF were considered independent variables, while 1-minute abdominal test was the moderating variable. Johnson-Newman technique was applied to establish the moderation point. Analyzes were adjusted for sex, age, and APHV. Data analysis was performed with the SPSS v.24.0, the moderation models in the PROCESS macro for SPSS, and the significance level was *p* ≤ 0.05.

## Results

A total of 62 adolescents participated in the study, with a mean age of 14.3 ± 2.0 years, being 31 boys (14.4 ± 2.1 years) and 31 girls (14.2 ± 2.0 years; *p* = 0.67). The mean APHV was 13.0 ± 0.9 years, with significant differences between boys (13.7 ± 0.7 years) and girls (12.3 ± 0.5 years; *p* = 0.001). The results of the categorization of the sample according to sex of the anthropometric variables, body composition, CMRF, and 1-minute abdominal test (ABD) are presented in Table 1.

**Table 1:** Anthropometry, body composition, cardiometabolic risk factors and 1-minute abdominal test according to sex

Variables	All (*n* = 62)	Boys (*n* = 31)	Girls (*n* = 31)	F	p
**Anthropometry**					
Height (cm)	162.2 ± 9.2	165.9 ± 9.4	158.5 ± 7.4	673.1	**0.001**
Body Mass (kg)	71.0 ± 13.5	73.6 ± 12.8	68.4 ± 13.9	9.04	**0.004**
Body Mass Index (kg·m^-2^)	27.0 ± 4.6	26.9 ± 4.8	27.1 ± 4.5	0.59	0.440
Body Mass Index z-score	1.90 ± 1.0	1.92 ± 1.2	1.89 ± 0.8	0.12	0.729
Triponderal Mass Index (kg·m^-3^)	16.7 ± 3.1	16.3 ± 3.3	17.1 ± 2.9	6.30	**0.015**
Abdominal Circumference (cm)	91.6 ± 11.9	92.5 ± 12.8	90.7 ± 11.0	0.24	0.622
Waist Circumference (cm)	83.3 ± 10.3	85.3 ± 10.7	81.3 ± 9.7	0.89	0.347
Waist-to-Height Ratio	0.51 ± 0.07	0.52 ± 0.08	0.51 ± 0.06	2.48	0.121
**Body composition**					
Fat Mass (kg)	23.8 ± 8.8	22.6 ± 9.5	25.1 ± 8.0	0.47	0.829
Fat-Free Mass (kg)	47.1 ± 8.1	51.0 ± 7.4	43.3 ± 6.9	48.34	**< 0.001**
**Clinics and metabolic**					
Systolic Blood Pressure (mm Hg)	114.7 ± 12.1	118.9 ± 12.2	110.5 ± 10.7	18.08	**0.001**
Diastolic Blood Pressure (mm Hg)	66.9 ± 8.9	68.1 ± 10.4	65.8 ± 7.1	6.40	**0.011**
Mean Blood Pressure (mm Hg)	82.8 ± 9.1	85.0 ± 10.1	80.7 ± 7.4	12.54	**0.001**
HDL-c (mg·dL^-1^)	50.6 ± 9.6	50.9 ± 8.6	50.4 ± 10.6	0.34	0.876
LDL-c (mg·dL^-1^)	87.5 ± 26.6	89.0 ± 28.7	86.1 ± 24.7	0.02	0.957
Triglycerides (mg·dL^-1^)	102.1 ± 53.0	94.6 ± 53.6	109.7 ± 52.1	0.01	0.884
Adiponectin (µg·mL^-1^)	6.85 ± 3.8	6.95 ± 3.8	6.76 ± 3.9	0.39	0.444
**1-minute abdominal test**					
ABD (repetitions)	24.5 ± 11.7	26.8 ± 13.6	22.2 ± 9.0	4.83	**0.032**

HDL-c, high density lipoprotein cholesterol; LDL-c, low density lipoprotein cholesterol; ABD, 1-minute abdominal test; bold, statistical significance

Moderation analyses of 1-minute abdominal test in the relationship between anthropometric variables, body composition, CMRF, and adiponectin are represented in Table 2. Inverse interactions were found for adiposity (BMI, BMI-z, TMI, AC, WC, WHtR), body composition (FM, FFM) and CMRF (SBP, DBP, MBP, TG) versus 1-minute abdominal test with adiponectin concentration, demonstrating that abdominal test is a moderator in these relationships.

**Table 2:** Moderation of 1-minute abdominal test in the relationship between cardiometabolic risk factors and adiponectin concentration

	Adiponectin (µg·mL^-1^)					Adiponectin (µg·mL^-1^)		
	β	CI (95%)	p			β	CI (95%)	p
ABD	0.889	(0.386; 1.392)	0.001		ABD	0.736	(0.263; 1.208)	0.003
BMI (kg·m^-2^)	0.733	(0.228; 1.239)	0.005		FFM (kg)	0.302	(-0.003; 0.607)	0.052
ABD x BMI	-0.034	(-0.053; -0.014)	**0.001**		ABD x FFM	-0.014	(-0.024; -0.004)	0.005
ABD	0.231	(0.060; 0.402)	0.009		ABD	1.026	(0.073; 1.978)	0.035
BMI-z	2.728	(0.173; 5.282)	0.037		SBP (mm Hg)	0.183	(-0.054; 0.421)	0.128
ABD x BMI-z	-0.122	(-0.206; -0.038)	**0.005**		ABD x SBP	-0.009	(-0.017; 0.000)	**0.045**
ABD	0.558	(0.097; 1.018)	0.019		ABD	1.009	(0.266; 1.752)	0.009
TMI (kg·m^-3^)	0.684	(-0.079; 1.447)	0.078		DBP (mm Hg)	0.371	(0.047; 0.695)	0.025
ABD x TMI	-0.034	(-0.079; -0.004)	**0.027**		ABD x DBP	-0.015	(-0.027; -0.003)	**0.012**
ABD	1.429	(0.693; 2.164)	< 0.001		ABD	1.188	(0.300; 2.077)	0.010
AC (cm)	0.357	(0.145; 0.570)	0.001		MBP (mm Hg)	0.336	(0.022; 0.649)	0.036
ABD x AC	-0.016	(-0.024; -0.007)	**< 0.001**		ABD x MBP	-0.014	(-0.025; -0.003)	**0.003**
ABD	1.434	(0.718; 2.149)	< 0.001		ABD	-0.198	(-0.706; 0.310)	0.439
WC (cm)	0.353	(0.124; 0.582)	0.003		HDL-c (mg·dL^-1^)	-0.149	(-0.431; 0.133)	0,294
ABD x WC	-0.017	(-0.026; -0.009)	**< 0.001**		ABD x HLD-c	0.005	(-0.005; 0.015)	0.310
ABD	0.798	(0.129; 1.466)	0.020		ABD	-0.089	(-0.398; 0.221)	0.568
WHtR	26.990	(8.678; 62.658)	0.135		LDL-c (mg·dL^-1^)	-0.013	(-0.112; 0.085)	0.790
ABD x WHtR	-1.568	(-2.923; -0.213)	**0.024**		ABD x LDL-c	0.002	(-0.002; 0.005)	0.351
ABD	0.388	(0.147; 0.628)	0.002		ABD	0.274	(0.058;01.490)	0.014
FM (kg)	0.377	(0.095; 0.659)	0.010		TG (mg·dL^-1^)	0.056	(0.001; 0.110)	0.047
ABD x FM	-0.017	(-0.028; -0.007)	**0.002**		ABD x TG	-0.002	(-0.005; 0.000)	**0.026**

ABD, 1-minute abdominal test; BMI, body mass index; BMI-z, z-score BMI; TMI, triponderal mass index; AC, abdominal circumference; WC, waist circumference; WtHR, waist-to-height ratio; FM, fat mass; FFM, fat-free mass; SBP, systolic blood pressure; SBP, diastolic blood pressure; MBP, mean blood pressure; HDL-c, high density lipoprotein; LDL-c, low density lipoprotein; TG, triglycerides. All analyses were adjusted for sex. age and age of peak height velocity; bold, statistical significance

**Fig. 1:**
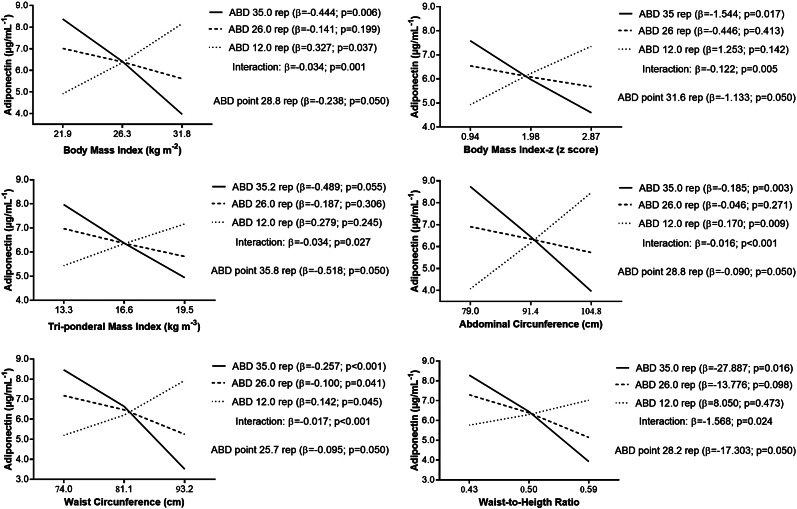
Relationship between adiposity and adiponectin concentration (µg·mL^-1^) according to levels of 1-minute abdominal test (ABD). Analyses were adjusted for sex, age, and somatic maturation

**Fig. 2:**
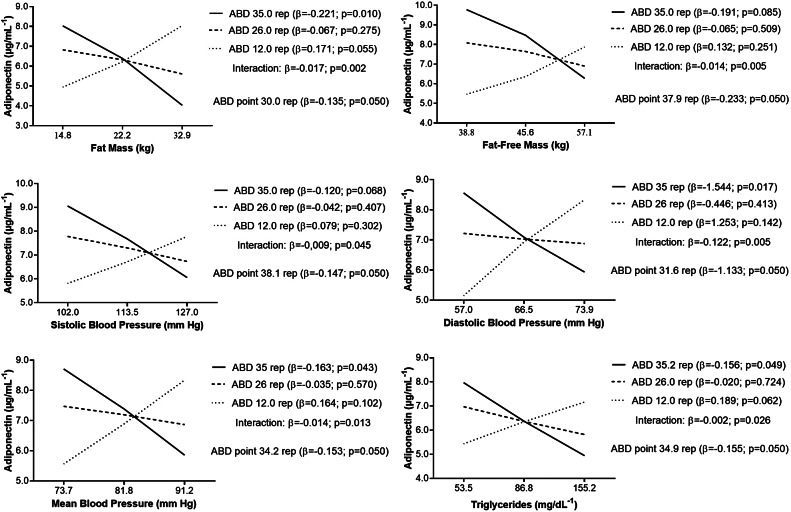
Relationship between cardiometabolic risk factors and adiponectin concentration (µg·mL^− 1^) according to levels of 1-minute abdominal test (ABD). Analyses were adjusted for sex, age, and somatic maturation

Results indicated that higher levels of BMI, BMI-z, AC, WC, WHtR, FM, DBP, MBP, and TG were associated with lower adiponectin concentration in adolescents with low ABD (35 rep), while adolescents with low ABD (12 rep) and high BMI, AC and WC had higher adiponectin (Figs. 1 and 2). Association was observed in those with low ABD, as adolescents with medium and high ABD, the association was no longer observed, suggesting that those who achieved more than 25 rep (for WC), 28 rep (for BMI; AC; WHtR), 30 rep (for FM), 31 rep (for BMI-z; DBP); 34 rep (for MPB; TG), 35 rep (for TMI), 37 rep (for FFM) and 38 rep (for SBP) on the abdominal test.

## Discussion

Studies have sought to verify the independent effects of MF on physical and cardiometabolic health in adolescents, and its role has been recognized due to its benefits, since an inverse association as cardiometabolic risk has been determined [17, 26]. However, few studies have investigated the relationship with inflammatory markers [17, 27], and investigations showed that adiponectin was inversely associated with MF [13, 15], while others found no relationship [19, 20].

Some results of our survey were different from what was expected, a factor that leads to an investigation focused on the paradoxical function that adiponectin can play, as well as when it is necessary to increase its secretion for metabolic protection. Anti-inflammatory effect can be crucial according to metabolic demand, perhaps its concentration is reduced in adolescents who do not need its protection, MF can play a decisive role in adiposity and in the inflammatory process [18, 28]. However, due to the inconsistencies of the findings to date, it was necessary to better understand the relationship between 1-minute abdominal test and adiponectin in adolescents, and to our knowledge, little is known about the moderating role of 1-minute abdominal test in the relationship between adiposity, CMRF and the adiponectin concentration.

The 1-minute abdominal test (ABD) is a moderator in the relationship between adiposity, body composition and adiponectin, ABD modifies the relationship between the low, medium, and high terciles of BMI, BMI-z, AC, WC, WHtR and the FM. Observed that in adolescents with low ABD, the 1-minute abdominal test may not neutralize the harmful effects caused by high BMI, AC, and WC, even with higher adiponectin, which probably provided metabolic protection. On the other hand, it was found that in adolescents with high ABD, there is no need for the anti-inflammatory effects attributed to the higher adiponectin for metabolic protection.

Therefore, our findings suggest that adolescents with medium and high ABD may be protected from the risks associated with obesity and, as a result, do not need high concentrations of adiponectin. Based on our analyses, we provided the level of ABD necessary for this protection to exist for adolescents, demonstrating that they should reach at least the threshold point of 25 repetitions in the abdominal test. Results found in our study, which showed an inverse interaction of 1-minute abdominal test in the relationship between adiposity, body composition and adiponectin concentration, agree with previous studies in adolescents [13, 15].

Our findings are consistent with those Agostinis-Sobrinho et al. [13] who reported an inverse and independent association of adiponectin and MF. Eutrophic adolescent with high MF had low adiponectin compared to adolescents with low MF, and overweight adolescents with high MF had lower adiponectin than eutrophic adolescents with low MF. Martinez-Gomes et al. [15] found MF score was independently and inversely associated with adiponectin concentration, and further analyzes revealed lower concentrations of adiponectin in the healthy group when compared to the medium-healthy and unhealthy groups. Furthermore, MF moderated the relationship between waist circumference and cardiometabolic risk score in eutrophic girls, conferring a protective effect against metabolic risk [18].

Protective role of MF was found on CMRF in children and adolescents of both sexes, and the effect of this association seems to be mediated by adiposity levels in children of both sexes and in adolescent boys [29]. Evidence has shown that MF was inversely and independently associated with cardiometabolic risk factors in adolescents, exerting a protective role and could attenuate the deleterious effects of obesity on cardiometabolic risk [30]. It should be noted that good levels of MF in the early stages of life showed protection in cardiometabolic health in adulthood [31].

We found that 1-minute abdominal test moderates the relationship between cardiometabolic risk and adiponectin, noting that ABD modifies the relationship between the low, medium, and high terciles of SBP, MBP, and TG. We observed that in adolescents with high ABD, there may be no need for higher concentrations of adiponectin for metabolic protection. Therefore, our findings suggest that adolescents with medium and high ABD may be protected from the risks related of SBP, MBP, and TG. In addition, we provided the level necessary for the protection, demonstrating that with the threshold point of 34 repetitions in the abdominal test.

In the adult population, adiponectin is inversely associated with risk factors for cardiovascular diseases, including dyslipidemia and arterial hypertension [32, 33]. In the pediatric population, evidence has suggested that adiponectin is inversely associated with SBP [34], DBP [34] and high blood pressure, regardless of obesity [35]. Furthermore, it is inversely associated with TG [36] and has a protective effect against atherosclerosis, inhibiting the expression of inflammatory cytokines in the vascular endothelium, modulating the endothelial inflammatory response [37].

Therefore, low levels of MF are considered an independent risk factor and predictor for cardiovascular diseases, morbidity, and mortality in adults [38], and an important health marker for the child and adolescent population [17]. The MF can play a determining role, demonstrating that the deleterious effects attributed to CMRF can be mitigated for the 1-minute abdominal test, reinforcing the role of physical fitness in physical health and cardiometabolic in adolescents. Therefore, due to its health benefits, the current physical activity guidelines for children and adolescents recommend at least three days of muscular strengthening activities [39], requiring incentives and interventions that would help adolescents to practice more regular physical exercise, to improve physical fitness and reduce adiposity.

However, we are conscious that the sample size used in the present study does not allow the generalization of the results, factors that must be analyzed with caution. Likewise, the cross-sectional design employed does not allow causality to be inferred. The measurement of adiponectin concentration was performed in its entirety, resulting from various tissues and not in its different isoforms, such as its high molecular weight oligomer (considered the biologically active form). Additional research corroborating the results presented in the current study would therefore be useful in to understand the role of different isoforms, as well as the role of adiponectin receptors.

## Conclusion

We conclude that 1-minute abdominal test may play an important role in the relationship between obesity and cardiometabolic risk. We found that abdominal test can confer a protective effect on adolescents with high levels of ABD, demonstrating the importance of maintaining body adiposity, cardiometabolic health and adequate levels of physical fitness from childhood and adolescence.

## Data Availability

The research dataset is not publicly available to preserve the privacy of the individuals. Data are available on request from the authors. Requests can be sent to the e-mail mctadiotto@gmail.com.

## Abbreviations

ABD: 1-minute abdominal test
AC: abdominal circumference
APHV: age at peak height velocity
BMI: body mass index
BMI-z: body mass index score z
DBP: diastolic blood pressure
FFM: fat-free mass
FM: fat mass
HDL-c: high density lipoprotein
LDL-c: low density lipoprotein
MBP: mean blood pressure
MF: muscular fitness
SBP: systolic blood pressure
TG: triglycerides
TMI: triponderal body index
WC: waist circumference
WHtR: waist-to-height ratio
